# Winter is not coming: The role of ClCNGC2 and ClCNGC20 in watermelon cold tolerance

**DOI:** 10.1093/plphys/kiaf312

**Published:** 2025-07-18

**Authors:** Sara Selma

**Affiliations:** Assistant Features Editor, Plant Physiology, American Society of Plant Biologists; VIB Center for Plant Systems Biology, 9052 Ghent, Belgium

Cold is a big threat to plant growth, disrupting vital physiological processes and thus decreasing crop productivity. Since seeking shelter is not an option for plants, they have evolved complex regulatory mechanisms to face cold stress. One of the key mechanisms in plant cold tolerance comprises the C-repeat binding factor (CBF) pathway, which activates downstream cold-responsive (COR) genes, thereby enhancing cold resilience (Park et al. 2015). The stress phytohormone jasmonate (JA) and its derivative, methyl jasmonate (MeJA), are characterized as pivotal signaling molecules against environmental stress. In particular, under cold conditions, JA biosynthesis and signaling are upregulated. Furthermore, exogenous MeJA application reduces cold damage in several species, including *Arabidopsis thaliana* (Arabidopsis), tomato, peach, and watermelon (Ali and Baek 2020). Although the mechanism by which jasmonates regulate cold tolerance in plants is not fully understood, one proposed mechanism involves the interaction between JAs and calcium (Ca²⁺) signaling. JAs can elevate cAMP levels, activating Ca²⁺ channels and promoting apoplastic Ca²⁺ influx (Lu et al. 2016). Ca²⁺ acts as a second messenger in cold signal transduction through Ca²-binding sensor proteins, activating downstream responses, and transcriptional regulatory cascades such as the CBF pathway (Liese and Romeis 2013).

The cyclic nucleotide-gated ion channels (CNGCs) are calcium-permeable channels that are able to regulate the Ca²⁺ influx during plant growth and development but also in response to environmental stresses (Dietrich et al. 2020). Several members of the CNGCs family have been identified in various species, such as Arabidopsis, rice, and tomato; however, the role of CNGCs in cold tolerance in other crop species is still not completely characterized.

Watermelon (*Citrullus lanatus*) is one of the top 5 most consumed fresh fruits globally, but due to its thermophilic nature, it is highly vulnerable to cold stress, which severely affects crop productivity (Rivero et al. 2001). Recently in *Plant Physiology*, (Guo et al. 2025) identified the CNGCs in watermelon that are involved in the Ca²⁺ influx in the cytoplasm during the MeJA-mediated response to cold stress.

As a first step, the effect of MeJA on watermelon cold tolerance was evaluated by comparing MeJA-pretreated seedlings with non-pretreated seedlings exposed to cold. Visually, the watermelon seedlings pretreated with MeJA and then exposed to 4 °C cold stress for 48 h showed decreased cold-induced damage. At the transcriptional level, although both treatments showed an increase in the expression of cold-responsive genes, MeJA-pretreated seedlings exposed to cold exhibited a 132.96%, 80.24%, and 121.81% increase in the expression of *ClCBF1*, *ClCBF2*, and *ClCOR47*, respectively, compared to seedlings exposed to cold alone, pointing to the role of MeJA in the cold resilience mechanisms.

Additionally, a virus-induced gene silencing (VIGS) approach was employed to generate watermelon plants with suppressed expression of watermelon jasmonic acid carboxyl methyltransferase (ClJMT), an enzyme responsible for the methylation of JA to MeJA (Qi et al. 2016). The partial silencing of *ClJMT* resulted in a decreased tolerance to cold stress and a less transcriptional activation of the *ClCBF1*, *ClCBF2*, and *ClCOR47* genes under cold conditions. These effects were alleviated upon the exogenous application of MeJA, confirming MeJA's essential role in the induction of cold-responsive genes.

To link the role of MeJA and the Ca²⁺ influx during cold response, the levels of Ca²⁺ were measured in vivo employing noninvasive microtest technology, a technique to measure several ions/molecules flows in intact biological samples. Both MeJA treatment and cold exposure caused a significant influx of Ca²⁺ into watermelon mesophyll cells. The results also show that MeJA stimulated the cold-induced influx of Ca²⁺, as plants pretreated with MeJA exhibited a 70.29% increase in Ca²⁺ influx compared to plants without MeJA pretreatment after cold shock. This effect was also confirmed through fluorescent imaging of the calcium ions in the cytoplasm of watermelon protoplasts. On the other hand, silencing the gene *ClJMT* (lowering MeJA levels) decreases the Ca²⁺ influx and the calcium ions in the cytoplasm. Similarly, CaCl₂ treatment improves cold tolerance in watermelon and activates the cold-responsive genes. Finally, the use of Ca²⁺ channel blockers (LaCl₃) and chelators (EGTA) suppresses MeJA-induced cold tolerance, highlighting the link between MeJA and Ca²⁺ influx during cold stress.

To further investigate the role of Ca²⁺ influx in the cold response, the authors investigated the role of the calcium-dependent protein kinases (CDPKs), which are known to decode calcium signals (Atif et al. 2019). In watermelon, 22 *ClCDPK* genes have been identified. The authors found that *ClCDPK8*, *ClCDPK10*, and *ClCDPK1* are significantly upregulated during the first hours upon cold stress combined with MeJA, while silencing *ClJMT* reduces the expression of these genes under cold conditions. These results suggest that the MeJA and cold-induced calcium influx are transduced by the CDPK genes in watermelon.

The authors also investigated the role of cyclic-nucleotide-gated channels (CNGC) in mediating the calcium influx. The authors identified 13 putative *ClCNGC* genes in watermelon. Of these, *ClCNGC2* and *ClCNGC20* showed higher and continuous responses to MeJA under cold stress. The silencing of the *ClCNGC2* and *ClCNGC20* genes resulted in a lower Ca²⁺ influx and in a significant drop in cold tolerance and the cold-responsive genes. However, the silencing of some other *ClCNGC* genes had minimal impact on cold tolerance. Additionally, to investigate the role of *ClCNGC2* and *ClCNGC20* in regulating Ca^2+^ influx and the cold, overexpressing Arabidopsis lines of *ClCNGC2* and *ClCNGC20* were generated. These Arabidopsis lines show an increased concentration of calcium ions in the cytoplasm and a greater Ca^2+^ influx in response to cold stimuli compared with wild-type plants. Upon cold stress, the overexpression lines show a higher upregulation of the genes of cold-responsive genes, *AtCBF1*, *AtCBF2*, and *AtCOR47*. Furthermore, the overexpression of *ClCNGC2* and *ClCNGC20* resulted in improved survival after freezing (−10 °C for 1 h), indicating the pivotal role of ClCNGC2 and ClCNGC20 in cold resilience.

Although it has been reported that certain CNGCs can form complexes to regulate Ca^2+^ influx, the results from protein-protein interaction experiments suggest that *ClCNGC2* and *ClCNGC20* do not physically interact. Interestingly, the expression of these genes shows a reciprocal dependency: silencing one suppresses the other. This reciprocal regulation supports a model (Figure.) where ClCNGC2 and ClCNGC20 jointly mediate MeJA-induced Ca²⁺ signaling and cold resistance.

**Figure.**
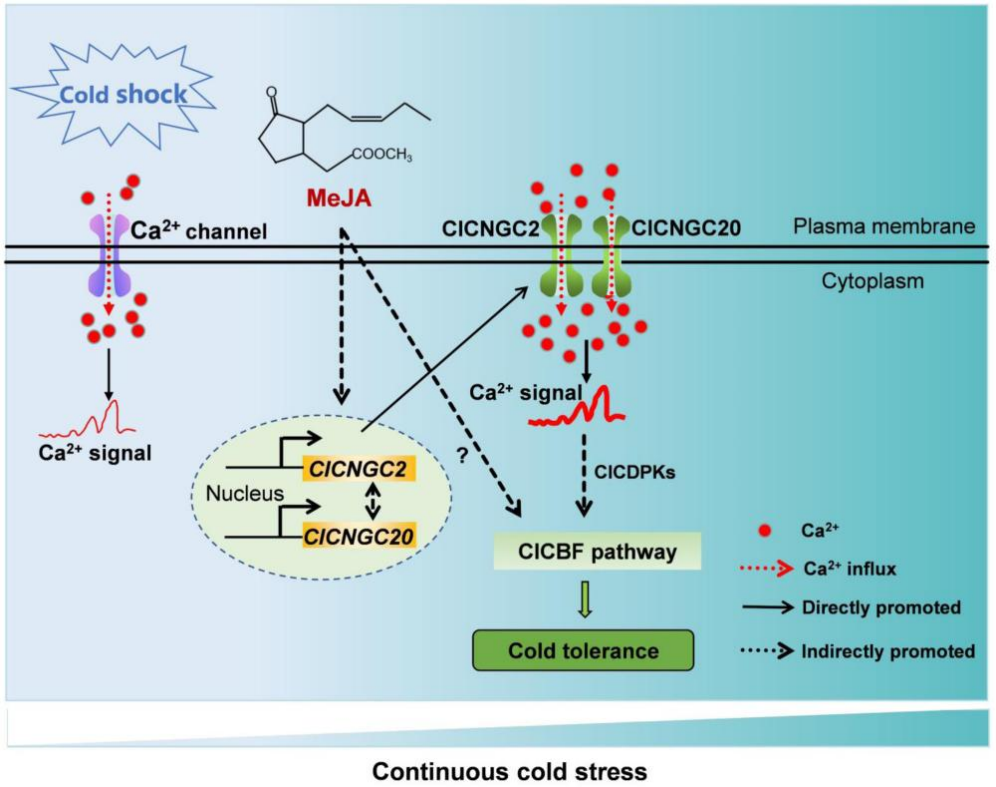
Molecular model of the MeJA-induced Ca^2+^ signaling through *ClCNGC2* and *ClCNGC20* in watermelon cold tolerance. Figure is from Guo et al. 2025.

In summary, the authors showed that MeJA enhances watermelon cold tolerance by activating calcium signaling via *ClCNGC2* and *ClCNGC20*, which triggers the CBF regulatory pathway and COR genes. These findings offer valuable targets for improving stress resilience in cold-sensitive crops. However, it is still not fully elucidated how the reciprocal regulation of *ClCNGC2* and *ClCNGC20* takes place under cold stress conditions. More research can be conducted to obtain a full picture of the molecular mechanism of MeJA-mediated cold resistance.
